# Supplementary material for: DiMB-RE: mining the scientific literature for diet-microbiome associations
Source: J Am Med Inform Assoc. 2025 Mar 27;32(6):998–1006. doi: 10.1093/jamia/ocaf054 (PMC12089768; doi:10.1093/jamia/ocaf054)
Supplement: ocaf054_Supplementary_Data [file ocaf054_supplementary_data.docx]

Appendix for *DiMB-RE: Mining the Scientific Literature for Diet-Microbiome Associations*

Gibong Hong, Veronica Hindle, Nadine M. Veasley, Hannah D. Holscher, and Halil Kilicoglu

A. [Queries for data collection from PubMed](#bookmark=id.gjdgxs)

B. [Annotation guidelines](#bookmark=id.1fob9te)

C. [PURE model for NER, trigger recognition, and RE](#_heading=h.ifjm1j99d6p)

D. [GPT* prompt for relation extraction (RE)](#bookmark=id.3dy6vkm)

E. [Experimental settings](#bookmark=id.1t3h5sf)

F. [Class-level results for NER, trigger recognition, and RE](#bookmark=id.3rdcrjn)

G. [PL-Marker error analysis](#bookmark=id.44sinio)

# Queries for data collection from PubMed

#1 (roseburia OR faecalibacterium OR eubacterium OR bacteroides OR akkermansia OR bifidobacteria OR prevotella OR lactobacillus OR ruminococcus OR firmicutes OR clostridium OR dialister OR oscillospira OR dorea OR blautia OR lachnopsira OR anaerostipes OR actinobacteria)
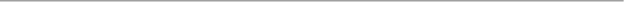
#2 AND (“short-chain fatty acids” (SCFA) OR acetate OR propionate OR butyrate OR “chenodeoxycholic acid” (CDCA) OR “deoxycholic acid” (DCA) OR “lithocholic acid” (LCA) OR phenols OR indoles OR valerate OR isovalerate OR isobutyrate OR “branch-chain fatty acids” (BCFA))
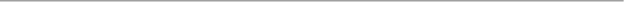
#3 AND (“visceral adiposity” OR “subcutaneous fat” OR “body weight” OR “body composition” OR “body mass index” (BMI) OR overweight OR obesity OR “metabolic disease” OR “cardiovascular disease” (CVD) OR atherosclerosis OR “nonalcoholic fatty liver disease” (NAFLD) OR “type 2 diabetes mellitus” (T2DM) OR inflammation OR hyperlipidemia OR glucose OR “glucose response” OR “glucose tolerance” OR “Hemoglobin A1C” (HbA1c) OR dyslipidemia OR “blood lipids” OR cholesterol OR triglycerides (TG) OR “blood pressure” OR “low-density lipoprotein cholesterol” (LDL-C) OR “high-density lipoprotein cholesterol” (HDL-C) OR “C-peptide” OR “Interleukin 6” (IL-6) OR “tumor necrosis factor alpha” (TNF-α) OR “high-sensitivity C-reactive protein” (hs-CRP) OR insulin OR “insulin resistance”)
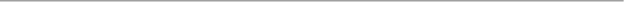
#4 AND (nuts OR legumes OR pulses OR “whole plant foods” OR fruit OR vegetable OR protein OR phytonutrient OR vitamin OR “dietary fiber” OR “dietary fibre” OR “fermentable fiber” OR “beta-glucan” OR “beta glucan” OR pectin OR arabinoxylan OR “guar gum” OR alginate OR “psyllium husk” OR inulin OR saccharide OR fructooligosaccharide (FOS) OR oligofructose OR galactooligosaccharide (GOS) OR xylooligosaccharide OR oligosaccharide OR polysaccharide OR cellulose OR “soy fiber” OR carbohydrate OR polyphenols OR “resistant maltodextrin” OR “resistant starch” OR prebiotic OR “prebiotic fiber” OR synbiotic OR “monounsaturated fatty acids” (MUFA) OR “polyunsaturated fatty acids” (PUFA) OR “saturated fatty acids” (SFA) OR “unsaturated fatty acids” (UFA) OR “Mediterranean diet”)
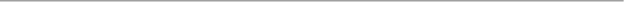
#5 NOT (probiotic NOT review NOT systematic review NOT meta-analysis NOT books NOT documents)

# Annotation guidelines

In this project, we will annotate nutrition/microbiome-related publications with entities of interest (e.g., foods, microbes, metabolites, diseases, physiological functions, populations, genes, enzymes, methods) and their relationships (e.g., food-microbe interactions). These annotations will be used as data for training text mining models that automatically recognize these entities and relationships in scientific publications.

In the first step of this annotation project, we will focus on publication titles and abstracts only. The annotation will be performed using the *brat* annotation tool^^[[1]](#footnote-1)^^. The annotation URL is: [URL]. Each annotator has their own annotation folder and should only annotate in that folder. To annotate, a username/password is needed. To login, hover over the document bar at the top of the page, which will display the login button on the right, as shown below. You can view annotations without logging in.


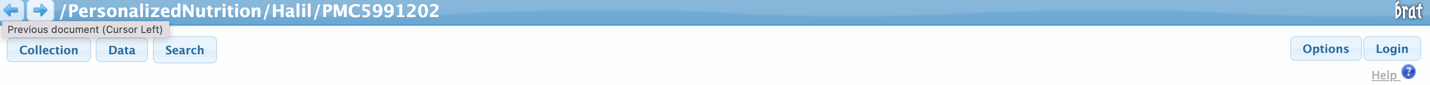


Each document in the annotation set corresponds to one publication. The PubMed Central ID of the publication is in the document title^^[[2]](#footnote-2)^^ (PMC5991202 in the screenshot above).

There are three steps to annotation:

1. Identify relevant entity mentions in the title/abstract and assign them entity types. Selecting the entity mention in text will bring up a dialog box where you can select the entity type. In the example below, text mention ‘Walnut’ is assigned the type Food from the list on the left.


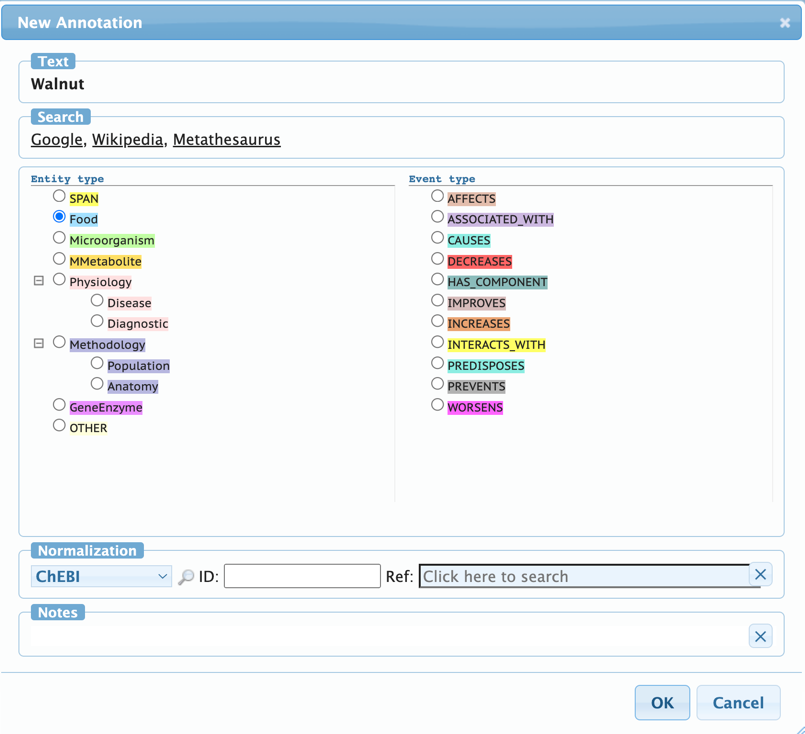


1. When a relationship is described between two entities, identify the word/phrase that indicates the relationship (i.e., its trigger) and assign the trigger a relation type from the list on the right.
   1. E.g., in the fragment “walnut consumption alters the gastrointestinal microbiota”, the trigger is “alters” which indicates an AFFECTS relationship.


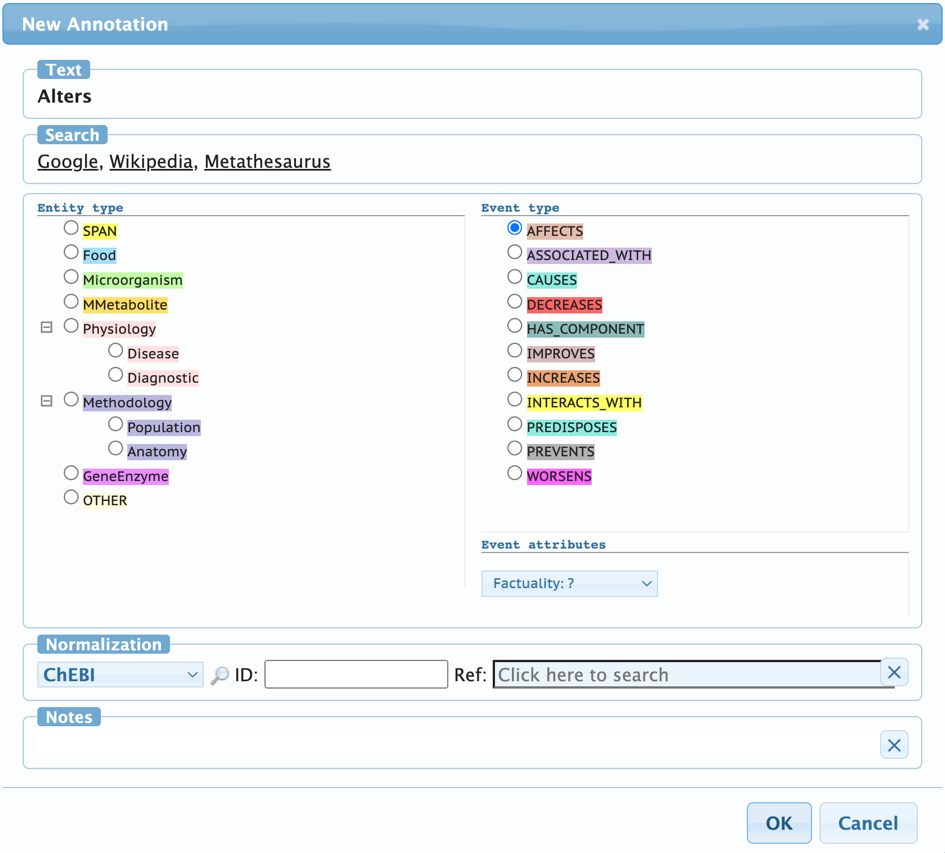


- 1. After this step, you should be able to view both the entity and trigger annotations.


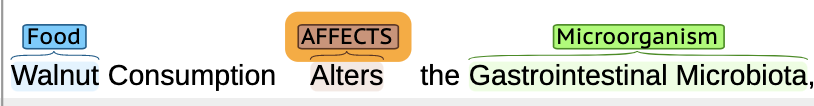


1. Link the trigger to the entity arguments and specify the role of the entity in the relationship (Agent or Theme).
   1. E.g., in the fragment “walnut consumption alters the gastrointestinal microbiota”, “walnut” is the Agent and “gastrointestinal microbiota” is the Theme^^[[3]](#footnote-3)^^ for “alters”. Move the cursor from “alters” to “walnut” and select Agent from the list, and to “gastrointestinal microbiota” to select Theme. When you move the cursor, an arrow will appear and you drag this cursor to the element you are linking to.


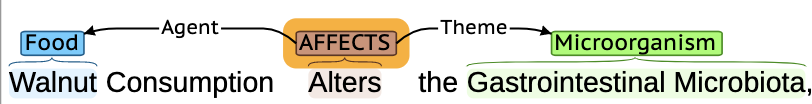


1. Assign a factuality value to the relationship (Factual, Probable, Possible, Doubtful, Negative, Unknown)
   1. In the example above, walnut-AFFECTS-gastrointestinal microbiota relationship is stated as factual. While assigning a relation label to the trigger (step 2), you can select the value Factual from the drop-down that appears under “Event attributes”. Note: This step is necessary only if the relationship is not stated as factual (because Factual is the default category).

We suggest annotating all relevant entity mentions in the publication title and abstract first. Then, identify relationships discussed between these entities and annotate the triggers and their links to the agent/theme. If the relationship is stated as uncertain (i.e., not factual), assign a factuality value.

**Entity Types:**

Note: Related entity types are grouped together in brat interface for ease of access as shown below.

- **Food:** Any substance that can be ingested by a living organism and metabolized into energy and body tissue.
  - **Nutrient:** Food components, such as carbohydrates, proteins, fats.
  - **DietPattern:** types of diets (Mediterranean diet, high protein, restricted diet, etc.)
- **Microorganism:** A microorganism, or microbe, is an organism of microscopic size, which may exist in its single-celled form or as a colony of cells.
  - **DiversityMetric:** A metric indicating the diversity of microorganisms (alpha diversity, etc.).
- **Chemical:** Compounds or substances of definite molecular composition. Here, we use it as a broad category for relevant chemicals that do not fit into more specific categories such as metabolite.
  - **Metabolite:** A chemical that is intermediate or end product of metabolism.
  - **Enzyme:** A substance that acts as a catalyst in living organisms (almost always a protein).
- **Gene**: A sequence of nucleotides in DNA that encodes the synthesis of a gene product, either RNA or protein.
- **Measurement:** A process/diagnostic measure to determine the composition, quantity, or concentration of a specimen and capture what is happening in a cell or an organism.
- **Physiology:** A process, activity, or state of the body.
  - **Disease:** A disordered process, activity, or state of the organism as a whole, of a body system or systems, or of multiple organs or tissues. We focus on specific diseases, like diabetes, cancer, rather than markers of diseases.
- **Methodology:** Study-level methodological characteristics of the publication. This may be a statistical analysis technique, instruments/biospecimens used, population or body part that is being studied (in which case use Population and Anatomy as relevant). Note that methodology entities will be study-level annotations (i.e., do not annotate them if they relate to the methodological characteristics of other studies).
  - **Population:** A group of individuals classified according to their sex, age, racial origin, social status, or some other cultural or behavioral attribute. Includes species other than humans, as well, such as mice, primates, etc. (UMLS)
  - **Biospecimen:** Denotes the type of sample used to characterize the microbiota/microbiome (e.g., gut, intestinal, gastrointestinal, colon, cecum, cecal, mucosa, oral) or the diagnostic outcome (e.g., serum, plasma, blood, saliva).

**Relation Types:**

Note that Methodology-related entities do not have any associated relationships and annotated at the article level (i.e., methodological aspects of the reported study only).

- **INTERACTS_WITH:** Acts, functions, or operates together with. The direction of the interaction may not be explicit.
  - Only takes Theme arguments. (Theme-INTERACTS_WITH-Theme)
  - Theme: Food, Microorganism, Chemical, Metabolite, Gene, Enzyme
- **INCREASES/DECREASES:** Make greater (or smaller) in size, amount, intensity, or degree. Similar to INTERACTS_WITH, but the direction is explicit.
  - Agent: Food, Physiology, Disease, Chemical, Metabolite, Microorganism, Gene, Enzyme
  - Theme: Chemical, Metabolite, Microorganism, Physiology, Gene, Enzyme, Measurement


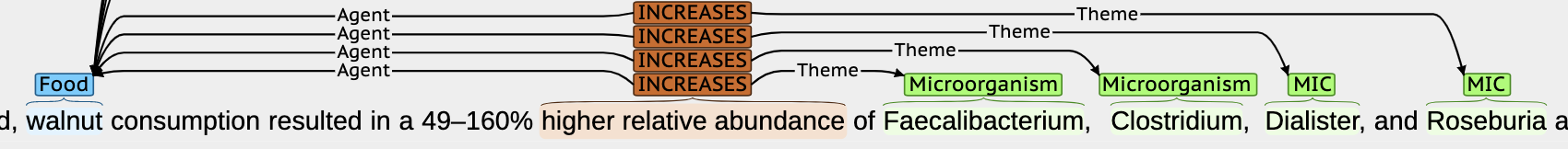


- **AFFECTS:** Produces a direct effect on. (UMLS) The nature of the effect is not explicit.
  - Agent: Food, Disease, Chemical, Metabolite, Microorganism, Physiology, Gene, Enzyme
  - Theme: Disease, Chemical, Metabolite, Microorganism, Physiology, Gene, Enzyme, Measurement


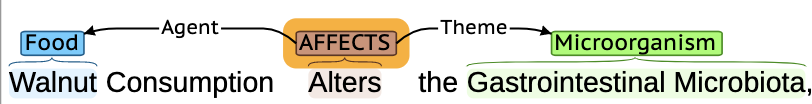


- **IMPROVES/WORSENS:** Similar to AFFECTS, but the nature of the effect is explicit.
  - Same Agent and Theme as AFFECTS.
- **CAUSES:** Brings about a condition or an effect. Implied here is that an agent, such as for example, a pharmacologic substance or an organism, has brought about the effect.
  - Agent: Food, Disease, Chemical, Metabolite, Microorganism, Physiology, Gene, Enzyme
  - Theme: Physiology, Disease
- **PREDISPOSES:** To be risk for a disorder, condition.
  - Same as CAUSES, except Disease cannot be an agent.
- **PREVENTS:** Stops, hinders or eliminates an action or condition. (UMLS)
  - Same as PREDISPOSES


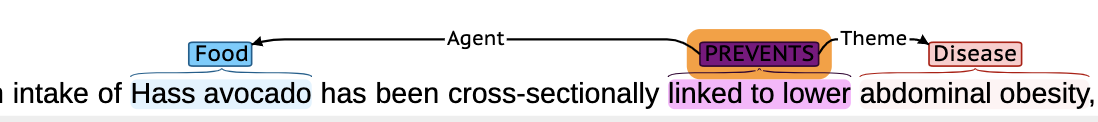


- **ASSOCIATED_WITH:** An unspecific relationship, a correlation, etc. It generally only takes Themes, but in some cases, Agent could be inferred too.
  - **POS_ASSOCIATED_WITH/NEG_ASSOCIATED_WITH:** Positive and negative correlation, respectively. Different from AFFECTS in that the relationship is not direct.
  - Agent/Theme: Food, Disorder, Chemical, Metabolite, Microorganism, Physiology, Gene, Enzyme, Measurement


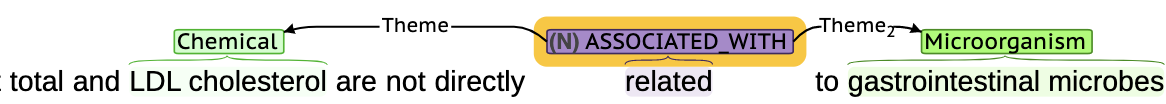


- **HAS_COMPONENT:** Nutrients, chemicals in food.
  - **Agent:** Food,
  - **Theme:** Food, Nutrient, Chemical, Microorganism


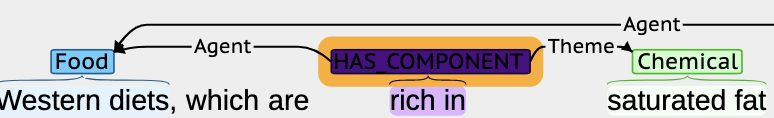


Factuality levels:

Factual – Probable – Possible – Doubtful – Negated can be thought of as a spectrum.

- **Factual:** The relationship is expressed as an assertion (e.g., ‘walnut consumption alters the gastrointestinal microbiota’). Factual is the default factuality category and does not need to be explicitly labeled.
- **Probable:** The relationship is expressed as less than certain, but likely. (‘e.g. ‘these results suggest that walnut consumption alters the gastrointestinal microbiota’).
- **Possible:** Less likely than probable, but still more likely than not. (e.g., ‘walnut consumption might alter the gastrointestinal microbiota’).
- **Doubtful:** More unlikely than likely. (e.g., ‘these findings do not lend support to the hypothesis that walnut consumption alters the gastrointestinal microbiota’)
- **Negated:** Asserted as a negative fact (e.g., ‘walnut consumption does not alter the gastrointestinal microbiota’).
- **Unknown:** No commitment made by the author regarding the certainty. (e.g., ‘we investigated whether walnut consumption alters the gastrointestinal microbiota’).

Guidelines/caveats:

- If brat does not allow you to select a particular entity as an argument of a relation due to its entity type, as a workaround, you can temporarily change its entity type to allow the relation BUT discuss this relation with others before proceeding, as it may point to a problem with the annotation scheme or the annotation itself.
- Do not annotate a trigger unless it indicates a relation that has BOTH an Agent and Theme argument.
- In the case of overlapping entities, typically the (larger) subsuming entity should be annotated. Subsumed entity could also be annotated if it is relevant to a particular relationship.
- Annotate the selected mention with the most specific type of entity/relationship you can identify. For example, entity type Metabolite are more specific than Chemical, relation type IMPROVES is more specific than AFFECTS.
- Following *informativeness* and *minimality* principles can ensure consistent annotations. Try to annotate the shortest span that indicates the relevant category and prefer a more specific/informative annotation over a less informative/generic annotation. (e.g., prefer ‘saturated fat’ over ‘fat’ only). Articles like ‘the’, ‘a’ can generally be excluded.
  - Avoid annotating generic terms, like bacterial taxa, bacterial gene. gene expression, host metabolism.
- Brat allows moving, deleting annotations, as well as adding fragments to it (non-contiguous spans). To perform these actions, double click on an existing annotation and select the appropriate button at the bottom. To remove entities that are included in relationships, remove the relationship trigger first.
- Annotate acronyms/abbreviations separately from the expanded form (e.g., in ‘short-chain fatty acids (SCFA)’, annotate ‘short-chain fatty acids’ and ‘SCFA’ as separate entities with the same type).
- Do not annotate coreferential expressions (e.g., pronouns like ‘it’, ‘they’ or noun phrases like ‘the treatments’ when they refer to entities described previously in the article). If these expressions license relationships, annotate as arguments the entities they refer to (i.e., antecedents) instead.
- Methodology and its subclasses are intended as article-level annotations. In other words, the specific Populations studied, Biospecimens and Study Design used should be annotated only at the appropriate granularity.
  - If the article reports a study about ‘beagle dogs’, but there are references to ‘dogs’ and perhaps ‘humans’, only annotate ‘beagle dogs’ because they are the topic of the study.
- When selecting for multi-word annotations, it is unfortunately very easy to exclude a letter or add a spurious letter with brat. Make sure your annotation boundaries are correct.

# PURE model for NER, trigger detection, and RE

We describe the PURE model for NER and RE (Zhong & Chen, 2021) below (Fig. 1). Similar to PL-Marker, PURE uses a pipeline approach with two independent encoder-only models for NER and RE. NER is formulated as span prediction. We obtain span representations from input tokens and feed them to a feedforward neural network to predict entity types. Specifically, we concatenate three hidden states from the first and the last token of a given span, as well as the embedding for the span length. The RE model finds the relation of every pair of entities predicted by the NER model. It handles each predicted entity pair independently and inserts typed markers around the subject and object entity spans (Soares et al., 2019). This input is fed to the encoder and the feedforward layer for relation classification. To fine-tune both the NER and RE models, we employ cross-entropy loss.

**Figure 1**. Overview of our model architecture based on PURE model [48]. We take a 3-step pipeline approach: 1) entity and trigger recognition (NER) 2) relation extraction (RE), and 3) factuality detection. Input to the model are individual sentences of the document. The model predicts entity mentions (with types) and their relations as well as the certainty levels of the predicted relations. In this example, the predicted relation is *PC enriched virgin olive oil-*decreases*-blood ox-ldl*, with the certainty level Factual.


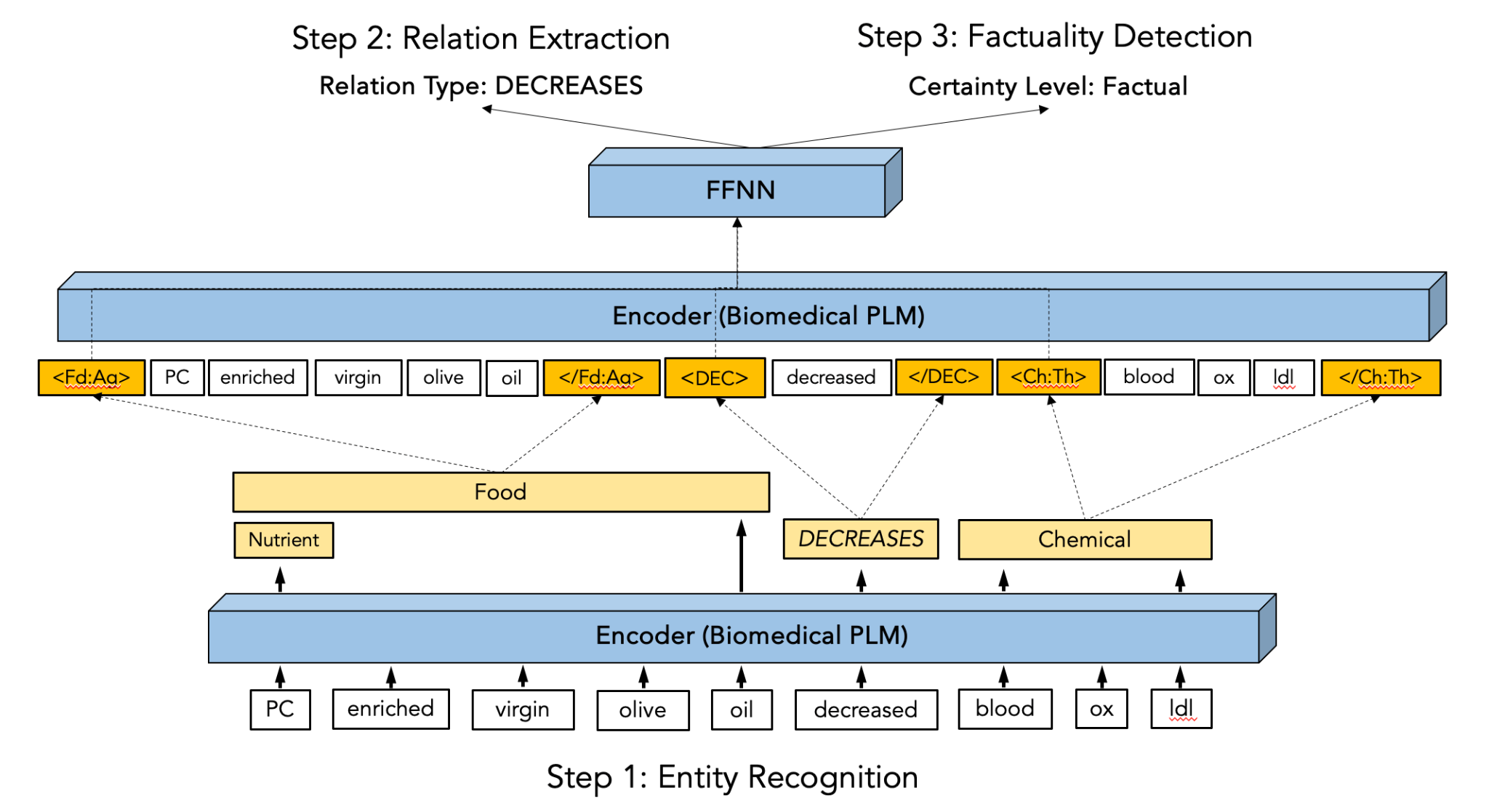


In using PURE for trigger detection, we trained separate feedforward layers and used different maximum span lengths for entities and triggers because trigger mentions are on average shorter than the entity mentions. To use trigger information for relation extraction, we concatenate the special start token (<trg>) with the entity pair to form the triple ([trg;entity1;entity2]) and the RE model trained to determine whether the triple is valid or not.

# GPT* prompt for RE

GPT* prompt used for RE tasks in DiMB-RE dataset is provided below. We articulate the task and dataset details in the task description part. Then, we add a fixed 1-shot example for every relation type in the guideline part. For selecting 1-shot example in this guideline part, we quantify the most frequent entity pair for each relation type then choose the example sentence including the entity type. Note that the demonstration part is only attached for 1-shot RE setup.

**[Task Description]**

You aim to extract relations (subject-relation-object triples) from biomedical articles on diet and microbiome. Subject and object refer to a pair of entity mentions described in text. Relations come from a pre-defined list. Please read carefully the following Guideline on the relevant relation types, their descriptions, and examples. The example provided with each relation type in the Guideline explains how the relation type between the entity pair could be inferred. Please use similar reasoning to identify the relation type that holds between the entity pair provided.
Note that entity roles (Subject as SUBJ, Object as OBJ) are assigned for each mention from a given pair. When you assume that there is an explicit direction for the relation based on the trigger information you’ve found, you should consider the direction from SUBJ to OBJ to make a final decision. Simply put, a relation can exist between A (role: Subject) and B (role: Object), while a relation between B (role: Subject) and A (role: Object) is invalid.

However, there are also bidirectional relation types, Interacts with, and (Positively/Negatively) Associated with. When you think the given entity pair has a bidirectional relation, then the relation between A (role: Subject) and B (role: Object) and the relation between B (role: Subject) and A (role: Object) can both be valid.
Each entity mention in the given pair is surrounded by the role and type information in the form of '<[ROLE]_START=[TYPE]> MENTION <[ROLE]_END=[TYPE]>'. In [ROLE] slots, SUBJ and OBJ could be placed, whereas for [TYPE] slots, different entity types can be placed.

**[Guideline]**

Guideline (Relation types and their descriptions are provided with examples)

- Interacts with: Acts, functions, or operates together with. The direction of the interaction may not be explicit.

<Example>

Sentence: Results suggested that GOS transport relies on a <SUBJ_START=Enzyme> permease <SUBJ_END=Enzyme> encoded by <OBJ_START=Gene> lacS <OBJ_END=Gene>, while a second unidentified protein may function as a galactoside transporter.

From the sentence above, the relation between 'permease' (role: Subject, type: Enzyme) and 'lacS' (role: Object, type: Gene) is ‘Interacts with’ because the relation is triggered by the mention ‘encoded’. In this context, it implies that the permease is involved with or functions together with the gene lacS in the process of GOS transport, which aligns with the definition of the relation type ‘Interacts with’. Also, the relation between 'permease' (role: Object, type: Enzyme) and 'lacS' (role: Subject, type: Gene) would also have the same relation type as the inferred relation is bidirectional.

- Increases: Make greater in size, amount, intensity, or degree. Similar to the definition of 'Interacts with', but the direction is explicit.

<Example>

…

- Has component: Relation where one entity is a constituent part of another. For example, Food entities can have Nutrients and Chemicals as components.

<Example>

Sentence: Supplementation of <OBJ_START=Nutrient> psyllium <OBJ_END=Nutrient> to <SUBJ_START=Food> milk replacer <SUBJ_END=Food> increased fermentation in the colon.

From the sentence above, the relation between ‘milk replacer’ (role: Subject, type: Food) and ‘psyllium’ (role: Object, type: Nutrient) is ‘Has component’ because the relation is triggered by the mention ‘Supplementation’. Because the ‘psyllium’ is added to the milk replacer, we can find that the relation between 'milk replacer' and 'psyllium' is categorized as 'Has component' based on the mention of supplementation.

Based on the guideline above, read the following sentence from a biomedical article and answer the questions.

**[1-shot demonstration]**

Sentence: Dietary supplementation with inulin - propionate ester or <SUBJ_START=Nutrient> inulin <SUBJ_END=Nutrient> improves <OBJ_START=Physiology> insulin sensitivity <OBJ_END=Physiology> in adults with overweight and obesity with distinct effects on the gut microbiota , plasma metabolome and systemic inflammatory responses : a randomised cross-over trial

Question 1: Following the above sentence, find out carefully whether there is a semantic relation between the entities ‘inulin’ and ‘insulin sensitivity’. A semantic relation exists if and only if there is an explicit syntactic relation between the entities. Note that about 90% of the time, an entity pair will be unrelated. The relation must be directional, where ‘inulin’ serves as a subject and ‘insulin sensitivity’ as an object. If the relation is directional but ‘insulin sensitivity’ is subject and ‘inulin’ is object, this should not be considered a relation. Terms and their acronyms or abbreviations should not be considered relations.

1. Relation exists

2. No relation

Question 2: If there is a relation, please select an option number and relation type below that best describes the relation of the given entity pair.

1. INCREASES

2. DECREASES

3. POS_ASSOCIATED_WITH

4. AFFECTS

5. PREVENTS

6. IMPROVES

7. ASSOCIATED_WITH

8. CAUSES

Question 3: Which of these options best describe the certainty level of the relation between the two entities?

1. Factual: It is used for relations that are expressed as an assertion or a fact.

2. Negated: It is used when relations are asserted as a negative fact.

3. Unknown: Less or no certainty about the relationship.

Please make sure that you should respond to Question 2 and 3 if you answer '1. There is a relation between the entity pair' for Question 1. If you answer A1: 2. No relation, then you should not answer Question 2 and 3.

When answering the questions, you should follow this format: 'A1: 1. Relation exists | A2: 1. RELATION-TYPE | A3: 2. FACTUALITY-LEVEL', or 'A1: 2. No relation'. Please use the bar sign to separate answers for each question.

Note that when there are no choice options in Question 2, you should only reply 2. No relation for Question 1.

A1: 1. Relation exists | A2: 6. IMPROVES | A3: 1. Factual

**[Test Input]**

Sentence: Effect of <SUBJ_START=Nutrient> vitamin E with therapeutic iron <SUBJ_END=Nutrient> supplementation on <OBJ_START=Physiology> iron repletion <OBJ_END=Physiology> and gut microbiome in U . S . iron deficient infants and toddlers : a randomized control trial

Question 1: Following the above sentence, find out carefully whether there is a semantic relation between the entities 'vitamin E with therapeutic iron' and 'iron repletion’. A semantic relation exists if and only if there is an explicit syntactic relation between the entities. Note that about 90% of the time, an entity pair will be unrelated. The relation must be directional, where ‘vitamin E with therapeutic iron’ serves as a subject and ‘iron repletion’ as an object. If the relation is directional but ‘iron repletion’ is subject and ‘vitamin E with therapeutic iron’ is object, this should not be considered a relation. Terms and their acronyms or abbreviations should not be considered relations.

1. Relation exists

2. No relation

Question 2: If there is a relation, please select an option number and relation type below that best describes the relation of the given entity pair.

1. INCREASES

2. DECREASES

3. POS_ASSOCIATED_WITH

4. AFFECTS

5. PREVENTS

6. IMPROVES

7. ASSOCIATED_WITH

8. CAUSES

Question 3: Which of these options best describe the factuality between the two entities?

1. Factual: It is used for relations that are expressed as an assertion or a fact.

2. Negated: It is used when relations are asserted as a negative fact.

3. Unknown: Less or no certainty about the relationship.

Please make sure that you should respond to Question 2 and 3 if you answer '1. There is a relation between the entity pair' for Question 1. If you answer A1: 2. No relation, then you should not answer Question 2 and 3.

When answering the questions, you should follow this format: 'A1: 1. Relation exists | A2: 1. RELATION-TYPE | A3: 2. FACTUALITY-LEVEL', or 'A1: 2. No relation'. Please use the bar sign to separate answers for each question.

Note that when there are no choice options in Question 2, you should only reply 2. No relation for Question 1.

**[END OF PROMPT]**

# Experimental settings

We tuned hyperparameters for NER and RE models on the validation set using greedy search. For LLM-based RE, we run GPT-4o-mini (*gpt-4o-mini-2024-07-18*) and GPT-4o (*gpt-4o-2024-08-06)* (OpenAI, 2024) using OpenAI API.

**BERT-based models**

For PL-Marker models, we mostly follow the optimal settings suggested from the original paper (Ye et al., 2022). For the NER model, we set learning rate as 2e-5, batch size as 8, maximum sequence length as 512, maximum length of levitated marker pairs as 256, maximum entity and trigger span length as 8. We trained the NER model for 50 epochs, evaluating its performance every 2 epochs and stopping the training early if the F_1_ score on the development set showed no improvement for five consecutive evaluations.

We also mostly follow the default settings for training the RE model; learning rate (2e-5), batch size (8), maximum sequence length (256), maximum length of object levitated marker pairs (16). Unlike the original settings (untyped solid marker for entity spans), we found that typed markers performed better for our dataset. Additionally, we didn’t use *inverse relations*, which assign pseudo inverse labels from object to subject entities, as it did not improve the performance for our dataset based on our preliminary experiments.

In the PURE NER model, as each span could have two different types of annotations (Entity or Trigger), the span representation from the output layer of NER model is fed into two multi-class classifiers (one for entities, one for triggers). Additionally, we extend the input sentence by setting a context window W=300 so that the NER module could use more contextual knowledge to identify entities or triggers. More precisely, as in the original model (Zhong & Chen, 2021), we augment the input with *(W - n)/2* words on each side of the input, given the input sentence with *n* words. The optimal parameters for the NER model were found to be: pre-trained model learning rate (1e-5), task learning rate (1e-3), context window (300), maximum entity span length (8), and maximum trigger span length (4). We use 4 NVIDIA Tesla V100-32GB GPUs, with batch size of 32 per device which reaches the maximum capacity of memory usage. We also set 200 training epochs, while doing evaluation after every 3 epochs and terminating the training session if there is no improvement in F_1_ within four consecutive evaluations for our development set.

For the PURE RE model, we use typed trigger representation, pre-trained model learning rate 3e-5, task learning rate 3e-5, and context window (100) were found optimal. For factuality detection, the optimal learning rate was 3e-5. We use Adam optimizer with a linear scheduler and a warm-up ratio of 0.1, following PURE. Here, we use the same 4 GPUs and the same batch size as we did in NER experiments. For RE training, we use 20 epochs for training, while doing evaluation every epoch and set the same termination policy as the NER training process.

**GPT-based models**

We used the first 100 samples from the validation set to optimize our prompting approach. RoBERTa-base (Liu et al., 2019) was used as the retrieval model and SimCSE (Gao et al., 2022) as the retrieval method. Temperature was set to 0 for deterministic results. For evaluation, we sampled a subset (10%) of our test set while keeping the proportion (90%) of NULL samples in the original test set. Table S1 presents the search range of each hyperparameter for the GPT prompt and models. For options style, we compare the 3-step multiple choices with NLI-based multiple choices. 3-step multiple choices first asks whether there is a relationship between the entities. If the response is positive, we then ask the relation and factuality label of the relation in a multiple-choice setting. For NLI-based prompt, we list all possible combinations of relation-factuality pairs with the multiple-choice settings. For example, if relation type INCREASES between entities A and B is possible, then three multiple choices are generated as *A increases B*, *A does not increase B*, and *A might increase B*, each denoting the factuality label of Fact, Negated, and Uncertain, respectively. We also add *No relation between A and B* as the last option. The 3-step multiple choice setting is illustrated in Section C.

To construct demonstrations, we select the SimCSE (Gao et al., 2021) to measure the semantic similarity between query sentences and candidates, then compare this method with random selection of demonstrations. We find that the demonstration retrieval based on SimCSE performs much better than simple random selection. Regarding the retrieval model, we use three different models: supervised-SimCSE-RoBERTa-base (Gao et al., 2021), BioSimCSE with BioLinkBERT-base model (Kanakarajan et al., 2022), and BiomedBERT-based sentence-transformer^^[[4]](#footnote-4)^^.

**Table S1.** The search space of GPT-based RE hyperparameters. Other hyperparameters which are not mentioned in this table simply follow the default setting of OpenAI API.

| **Hyperparameter** | **Search Range** | **Used** |
| --- | --- | --- |
| Options style | {3-step Multiple Choice, NLI} | 3-step Multiple Choice |
| Guideline | {Yes, No} | Y |
| Example for Guideline | {0, 1} | 1 |
| # of Demos | {0, 1, 2} | 1 |
| Retrieval Method | {SimCSE, Random} | SimCSE |
| Retrieval Model | {RoBERTa-base, BioMedBERT-base, PubMedBERT-base} | RoBERTa-base |
| Frequency_threshold | {3, 6} | 3 |
| Temperature | - | 0 |
| Top_p | - | 1 |
| Max_tokens | - | 4096 |
| Seed | - | 42 |

**Computation time and environmental impact**

For PL-Marker-related training, we iterated 50 training processes, which take 2 hours with 1 Tesla V100-32GB on average. We expect that training for PL-Marker resulted in around 0.5kg CO_2_e (Carbon dioxide equivalents) per training, 25kg in total. For PURE-related experiments, we ran 180 training processes, which take 2 hours with 4 Tesla V100-32GB on average. We estimate that this process resulted in around 1.35kg CO_2_e (Carbon dioxide equivalents) per training, 243kg in total. We used Green Algorithms calculator (Lannelongue et al., 2021) as a tool to compute environmental impact for the BERT-based fine-tuned models.

It is difficult to accurately estimate the environmental impact for GPT-4o and GPT-4o-mini, as OpenAI does not publicly release the relevant information including its model size (Li et al., 2024, Fu et al., 2024). We used the EcoLogits Calculator (<https://huggingface.co/spaces/genai-impact/ecologits-calculator>) to get an estimate for GPT-4o inference: 0.23kg CO_2_e per inference, 111kg in total. This tool does not provide estimates for GPT-4o-mini, but this is expected to be lower than GPT-4o.

# PURE model results

The performances of the PURE-based models are presented in Table S2. The training data includes Results sections in addition to titles and abstracts. The models yield higher precision than recall. Compared to strict evaluation, NER F_1_ increased by 5.9 percentage points and trigger recognition F_1_ by 4.8 points in relaxed evaluation. NER performance is higher than trigger detection performance. For individual entity types, the performance ranges from 0.908 for Microorganism to 0.547 for DietPattern. Among trigger types, we obtained the highest F_1_ score for neg_associated_with (0.889). The model was unable to recognize several trigger types with few training examples (e.g., interacts_with, predisposes, worsens).

The RE model yields 0.371 F_1_ score in strict evaluation and 0.409 in relaxed evaluation. The end-to-end model yields 0.356 F_1_ score in strict evaluation. The highest performing relation types are increases, has_component, and decreases, while the model fails for some relatively infrequent labels in the dataset (e.g., interacts_with, worsens).

**Table S2.** PURE-model performance on the test set. We provide mean performances over five training runs with different seeds, along with 95% CIs based on bootstrap sampling (in square brackets).

|  | | **Precision** | **Recall** | **F_1_** |
| --- | --- | --- | --- | --- |
| NER | Strict | 0.777 [0.774-0.779] | 0.745 [0.742-0.747] | 0.760 [0.759-0.761] |
|  | Relaxed | 0.852 [0.851-0.854] | 0.788 [0.784-0.792] | 0.819 [0.817-0.822] |
| Trigger detection | Strict | 0.691 [0.685-0.700] | 0.631 [0.628-0.636] | 0.660 [0.657-0.663] |
|  | Relaxed | 0.742 [0.736-0.749] | 0.678 [0.673-0.683] | 0.708 [0.706-0.710] |
| RE | Strict | 0.416 [0.404-0.424] | 0.336 [0.324-0.346] | 0.371 [0.364-0.378] |
|  | Relaxed | 0.458 [0.451-0.466] | 0.370 [0.357-0.383] | 0.409 [0.403-0.416] |
| Factuality detection | | 0.955 [0.948-0.963] | 0.415 [0.397-0.433] | 0.578 [0.560-0.592] |
| End-to-end | Strict | 0.399 [0.387-0.409] | 0.322 [0.309-0.333] | 0.356 [0.348-0.364] |
|  | Relaxed | 0.440 [0.431-0.447] | 0.355 [0.344-0.367] | 0.393 [0.387-0.399] |

Performance comparison of PURE and PL-Marker models in F1 score is provided in Table S3. PL-Marker outperforms PURE for all tasks, ranging from 1.1 percentage points in trigger detection (strict) to 11 points in RE and end-to-end pipeline (relaxed).

**Table S3.** Comparison of PURE and PL-Marker models in F1 score on test set. We provide mean performances over five training runs with different seeds, along with 95% CIs based on bootstrap sampling (in square brackets).

|  | | | **F_1_** |
| --- | --- | --- | --- |
| NER | Strict | PURE | 0.760 [0.759-0.761] |
|  |  | PL-Marker | 0.800 [0.793-0.808] |
|  | Relaxed | PURE | 0.819 [0.817-0.822] |
|  |  | PL-Marker | 0.862 [0.855-0.869] |
| Trigger detection | Strict | PURE | 0.660 [0.657-0.663] |
|  |  | PL-Marker | 0.671 [0.662-0.678] |
|  | Relaxed | PURE | 0.708 [0.706-0.710] |
|  |  | PL-Marker | 0.733 [0.724-0.738] |
| RE | Strict | PURE | 0.371 [0.364-0.378] |
|  |  | PL-Marker | 0.458 [0.445-0.472] |
|  | Relaxed | PURE | 0.409 [0.403-0.416] |
|  |  | PL-Marker | 0.519 [0.502-0.535] |
| Factuality detection | | PURE | 0.578 [0.560-0.592] |
|  |  | PL-Marker | 0.679 [0.670-0.686] |
| End-to-end | Strict | PURE | 0.356 [0.348-0.364] |
|  |  | PL-Marker | 0.445 [0.432-0.459] |
|  | Relaxed | PURE | 0.393 [0.387-0.399] |
|  |  | PL-Marker | 0.503 [0.488-0.518] |

# Class-level results for NER, trigger detection, and RE

Table S4 presents the NER performance of the best-performing model at the entity type level (PL-Marker NER model, including the Results sections in training).

**Table S4.** Test set performance of NER models at the entity type level (strict evaluation). We provide mean averages over 5 runs with different seeds, along with 95% CIs in the square brackets.

|  | **Precision** | **Recall** | **F1** |
| --- | --- | --- | --- |
| Chemical | 0.784 [0.779-0.790] | 0.774 [0.766-0.783] | 0.779 [0.774-0.785] |
| DietPattern | 0.480 [0.454-0.506] | 0.898 [0.819-0.958] | 0.625 [0.584-0.662] |
| Disease | 0.835 [0.823-0.849] | 0.882 [0.878-0.884] | 0.858 [0.850-0.866] |
| DiversityMetric | 0.565 [0.541-0.590] | 0.667 [0.667-0.667] | 0.611 [0.597-0.626] |
| Enzyme | 0.938 [0.890-0.977] | 0.933 [0.893-0.973] | 0.934 [0.915-0.953] |
| Food | 0.855 [0.837-0.881] | 0.708 [0.677-0.746] | 0.774 [0.751-0.797] |
| Gene | 0.511 [0.467-0.549] | 0.889 [0.889-0.889] | 0.647 [0.616-0.678] |
| Measurement | 0.774[0.750-0.799] | 0.776 [0.764-0.788] | 0.775 [0.761-0.791] |
| Metabolite | 0.822 [0.798-0.840] | 0.847 [0.821-0.870] | 0.834 [0.810-0.853] |
| Microorganism | 0.937 [0.930-0.946] | 0.915 [0.908-0.923] | 0.926 [0.921-0.933] |
| Nutrient | 0.820 [0.798-0.842] | 0.849 [0.838-0.864] | 0.834 [0.821-0.851] |
| Physiology | 0.687 [0.679-0.696] | 0.720 [0.712-0.732] | 0.703 [0.695-0.711] |
| MICRO-AVERAGE | 0.787 [0.778-0.797] | 0.814 [0.806-0.820] | 0.800 [0.793-0.808] |

Table S5 presents the trigger recognition performance of the best-performing model at the trigger type level (PL-Marker NER model with entities and triggers including the Results sections in training).

**Table S5**. Test set performance of the best trigger recognition model at the type level (strict evaluation). We provide mean averages over 5 runs with different seeds, along with 95% CIs in the square brackets.

|  | **Precision** | **Recall** | **F1** |
| --- | --- | --- | --- |
| AFFECTS | 0.644 [0.598-0.686] | 0.688 [0.658-0.709] | 0.665 [0.625-0.695] |
| ASSOCIATED_WITH | 0.507 [0.455-0.565] | 0.600 [0.570-0.630] | 0.548 [0.509-0.586] |
| CAUSES | 0.733 [0.700-0.750] | 0.467 [0.400-0.500] | 0.569 [0.507-0.600] |
| DECREASES | 0.741 [0.737-0.748] | 0.810 [0.800-0.819] | 0.774 [0.767-0.782] |
| HAS_COMPONENT | 0.579 [0.546-0.619] | 0.629 [0.614-0.643] | 0.601 [0.588-0.616] |
| IMPROVES | 0.494 [0.460-0.525] | 0.522 [0.489-0.561] | 0.507 [0.477-0.536] |
| INCREASES | 0.736 [0.709-0.760] | 0.811 [0.803-0.819] | 0.771 [0.755-0.790] |
| INTERACTS_WITH | 0.550 [0.500-0.650] | 0.467 [0.333-0.733] | 0.491 [0.400-0.674] |
| NEG_ASSOCIATED_WITH | 0.889 [0.889-0.889] | 0.800 [0.800-0.800] | 0.842 [0.842-0.842] |
| POS_ASSOCIATED_WITH | 0.613 [0.507-0.667] | 0.500 [0.500-0.500] | 0.546 [0.495-0.571] |
| PREDISPOSES | 0.933 [0.800-1.000] | 0.467 [0.333-0.600] | 0.593 [0.500-0.713] |
| PREVENTS | 0.288 [0.244-0.324] | 0.300 [0.233-0.333] | 0.293 [0.232-0.328] |
| WORSENS | 0.833 [0.633-1.000] | 1.000 [1.000-1.000] | 0.893 [0.787-1.000] |
| MICRO-AVERAGE | 0.652 [0.640-0.664] | 0.691 [0.682-0.698] | 0.671 [0.662-0.678] |

Table S6 presents the RE performance of the best-performing model at the relation type level. This model uses the PL-Marker NER model output (named entity and typed trigger recognition) and includes the Results sections in training.

**Table S6.** Test set performance of the RE model at the type level (strict evaluation).

|  | **Precision** | **Recall** | **F1** |
| --- | --- | --- | --- |
| AFFECTS | 0.383 [0.341-0.418] | 0.379 [0.339-0.409] | 0.381 [0.341-0.412] |
| ASSOCIATED_WITH | 0.170 [0.122-0.217] | 0.323 [0.277-0.362] | 0.219 [0.174-0.267] |
| CAUSES | 0.800 [0.667-0.933] | 0.333 [0.333-0.333] | 0.467 [0.444-0.489] |
| DECREASES | 0.580 [0.565-0.594] | 0.536 [0.516-0.554] | 0.557 [0.541-0.573] |
| HAS_COMPONENT | 0.533 [0.488-0.580] | 0.507 [0.473-0.541] | 0.518 [0.483-0.552] |
| IMPROVES | 0.452 [0.415-0.484] | 0.338 [0.307-0.373] | 0.386 [0.353-0.416] |
| INCREASES | 0.594 [0.562-0.635] | 0.554 [0.531-0.579] | 0.573 [0.546-0.609] |
| INTERACTS_WITH | 0.800 [0.533-1.000] | 0.500 [0.300-0.750] | 0.585 [0.389-0.794] |
| NEG_ASSOCIATED_WITH | 0.318 [0.263-0.373] | 0.365 [0.304-0.435] | 0.340 [0.282-0.398] |
| POS_ASSOCIATED_WITH | 0.379 [0.307-0.447] | 0.393 [0.313-0.447] | 0.382 [0.310-0.446] |
| PREDISPOSES | 0.871 [0.671-1.000] | 0.467 [0.244-0.622] | 0.592 [0.382-0.753] |
| PREVENTS | 0.567 [0.420-0.700] | 0.400 [0.314-0.514] | 0.468 [0.357-0.573] |
| WORSENS | 0.383 [0.283-0.533] | 0.240 [0.200-0.320] | 0.294 [0.233-0.400] |
| MICRO-AVERAGE | 0.463 [0.452-0.473] | 0.454 [0.435-0.471] | 0.458 [0.445-0.472] |

1. PL-Marker error analysis

We analyzed errors made by the PL-Marker model that uses trigger information. We reviewed 84 error cases from 7 randomly selected articles from the validation set in relaxed evaluation setting. We categorized errors into 8 types: Missed Entity, Missed Trigger, Entity Type Error, Trigger Type Error, Cross-sentence Relation, Complex Syntactic Structure, and Possible Inference. Counts, descriptions and specific examples of each error type are provided in Table S7.

**Table S7.** Error types and examples.

| **Error Type** | **FN** | **FP** | **TOTAL** | **Input sentence** | **Explanation** |
| --- | --- | --- | --- | --- | --- |
| Missed Entity | 3 | 0 | 3 | The non-responders to fibre treatment showed a decrease in microbiota diversity (Shannon and **Simpson diversity index** p-values of 0.0110 and 0.0255, respectively) after the intervention; where the reduction in short-chain fatty acids (SCFAs) producing bacterial genera such as Clostridium XIVa and Ruminococcus after dietary fibre treatment was the main difference. | NER module fails to identify the entity mention *Simpson diversity index* as DiversityMetric. |
| Missed Trigger | 18 | 10 | 28 | **Effect** of vitamin E with therapeutic iron supplementation on iron repletion and gut microbiome in U.S. iron deficient infants and toddlers: a randomized control trial | Trigger extraction module fails to identify the trigger mention *Effect* as AFFECTS. |
| Trigger Type | 5 | 8 | 13 | A Pilot Study of Human Milk to **Reduce** Intestinal Inflammation After Bone Marrow Transplant. | Trigger mention *Reduce* is tagged as DECREASES, instead of IMPROVES. IMPROVES is more appropriate here, due to the object *intestinal inflammation*, a health problem. |
| Entity Type | 10 | 10 | 20 | CONCLUSIONS : Consumption of **PE** decreased endotoxemia in overweight-obese individuals by reshaping the gut microbiota, mainly through the modulation of Faecalibacterium, Odoribacter, and Parvimonas. | *PE* is identified as Food instead of Nutrient. |
| Cross-sentence | 9 | 0 | 9 | Serum vitamin E concentration increased in **iron + vitamin E** group. *No change* over time was observed regarding serum **IL-4,** TNF-α or fecal calprotectin. | In the ground-truth relation ‘*iron + vitamin E* – AFFECTS (negated) - *IL-4*’, subject and object are located in different sentences. The model does not currently address such relations. |
| Complex Syntactic Structure | 0 | 3 | 3 | It suggested that the effect of **polysaccharides** cannot only be attributed to modulation of the gut microbiota, but also **associated** with the effect of microbial degradation on **GLPs** own activities. | The relation between *polysaccharides* and *GLPs* is likely wrongly predicted due to the prepositional phrase *the effect of microbial degradation on*. |
| Possible Inference | 0 | 8 | 8 | Fecal water from EVC001 - supplemented infants contains abundant indolelactate and B . infantis-derived **indole-3 -lactic acid** ( ILA ) upregulated immunoregulatory galectin-1 in Th2 and Th17 cells during polarization, providing a functional link between beneficial microbes and **immunoregulation** during the first months of life. | The relation between *indole-3-lactic acid* and *immunoregulation* can be inferred, but is not explicitly asserted and was not annotated. |
| TOTAL | 45 | 39 | 84 |  |  |

Notably, almost half of the errors originated from trigger related failures, and more than 75% of the total errors were caused by failures in NER and trigger detection. This finding, along with experiments with gold entities and triggers, highlights the critical importance of accurately identifying named entities and triggers for end-to-end performance.

Cross-sentence relations, which were not considered in this work, are a major cause of false negatives. Sentences with complex syntactic structures, such as coordination, negation, appositives, and other parenthetical expressions are typical in the corpus and are a considerable source of errors. We also identified some false positive cases, in which the extracted relation is not explicitly asserted and was not annotated, but could be inferred from the sentence. This type of error could be considered less severe.

In terms of nested entities, out of 17 pairs in the test set, both entities of the pair were extracted successfully once. Only the outer entity was extracted in 11 cases, only the inner entity in one case, and neither were extracted in 3 cases. The majority of the errors involved Food-Nutrient pairs.

# References

Fu, Z., Chen, F., Zhou, S., Li, H., & Jiang, L. (2024). LLMCO2: Advancing Accurate Carbon Footprint Prediction for LLM Inferences. *arXiv preprint* *arXiv*:2410.02950.

Gao, T., Yao, X., & Chen, D. (2021). SimCSE: Simple Contrastive Learning of Sentence Embeddings. In *Proceedings of the 2021 Conference on Empirical Methods in Natural Language Processing*, 6894–6910. <https://doi.org/10.18653/v1/2021.emnlp-main.552>.

Kanakarajan, K. R., Kundumani, B., Abraham, A., & Sankarasubbu, M. (2022). BioSimCSE: BioMedical Sentence Embeddings using Contrastive learning. In *Proceedings of the 13th International Workshop on Health Text Mining and Information Analysis (LOUHI)*, 81–86. <https://doi.org/10.18653/v1/2022.louhi-1.10>.

Lannelongue, L., Grealey, J., & Inouye, M. (2021). Green algorithms: quantifying the carbon footprint of computation. *Advanced science*, 8(12), 2100707.

Li, B., Jiang, Y., Gadepally, V., & Tiwari, D. (2024, November). Sprout: Green generative AI with carbon-efficient LLM inference. In *Proceedings of the 2024 Conference on Empirical Methods in Natural Language Processing* (pp. 21799-21813).

Liu, Y., Ott, M., Goyal, N., Du, J., Joshi, M., Chen, D., Levy, O., Lewis, M., Zettlemoyer, L., & Stoyanov, V. (2019). RoBERTa: A Robustly Optimized BERT Pretraining Approach (arXiv:1907.11692). *arXiv*. <http://arxiv.org/abs/1907.11692>.

OpenAI: Hurst, A., Lerer, A., Goucher, A.P., Perelman, A., Ramesh, A., Clark, A., Ostrow, A.J., Welihinda, A., Hayes, A., Radford, A. and Mądry, A., 2024. Gpt-4o system card. *arXiv preprint arXiv:2410.21276*. [https://arxiv.org/abs/*2410.21276*](https://arxiv.org/abs/2410.21276).

Soares, L.B., Fitzgerald, N., Ling, J., & Kwiatkowski, T*.* (2019). Matching the Blanks: Distributional Similarity for Relation Learning. In *Proceedings of the 57th Annual Meeting of the Association for Computational Linguistics*, 2895–2905.

Ye, D., Lin, Y., Li, P. and Sun, M. (2022). Packed Levitated Marker for Entity and Relation Extraction. In *Proceedings of the 60th Annual Meeting of the Association for Computational Linguistics (Volume 1: Long Papers)*, 4904-4917.

Zhong, Z., & Chen, D. (2021). A Frustratingly Easy Approach for Entity and Relation Extraction. In *Proceedings of the 2021 Conference of the North American Chapter of the Association for Computational Linguistics: Human Language Technologies,* 50-61.

1. For more information about brat, including examples, tutorials: <https://brat.nlplab.org/>. [↑](#footnote-ref-1)
2. Brat annotation tool removes all the formatting, so these IDs might be useful in locating the articles on PubMed Central to see its structure more clearly. [↑](#footnote-ref-2)
3. You can think of Agent and Theme as Subject and Object of a Verb. Note that relationships can be triggered by not only verbs, but also nominals, prepositions, adjectives. etc. [↑](#footnote-ref-3)
4. Model loaded from <https://huggingface.co/NeuML/pubmedbert-base-embeddings> [↑](#footnote-ref-4)
